# Critical Illness Polyneuropathy in Patients With Major Burn Injuries

**Published:** 2010-11-19

**Authors:** Queenie Chan, Karl Ng, John Vandervord

**Affiliations:** ^a^Severe Burns Injury Unit, Royal North Shore Hospital, St Leonards, Sydney, Australia; ^b^Department of Neurology and Clinical Neurophysiology, Royal North Shore Hospital, St Leonards, Sydney, Australia

## Abstract

**Objective:** Critical illness polyneuropathy in burn patients is an underreported condition. It is associated with high mortality rates and prolonged hospital stay and rehabilitation. This study aims to further define the cause and outcome of critical illness polyneuropathy following major burn injuries. **Methods:** A retrospective review of all burn patients with neuropathy that presented to Royal North Shore Hospital, Sydney, between the period of 2004 and 2009 was performed. The neurological findings, diagnostic processes, and outcomes were examined. End points such as duration on the ventilator, length of intensive care, and hospital stay were recorded. **Results:** There were 7 patients in total that exhibited abnormal neurological findings. Ages ranged from 17 to 43 years with all injuries sustained in flame burns. Mean total burnt surface area is 46%. There was no mortality in this series but all 7 had evidence of sepsis and multiorgan failure with an average 42 days spent on the ventilator. Clinical findings varied greatly. Five had involvement of nerves away from the site of burns. Upper-limb weakness tended to have a slower recovery. Examination and neurophysiologic studies were often hampered by pain and bandaging over burnt skin. **Conclusions:** Neurological manifestations of critical illness polyneuropathy are varied and cannot always be explained by direct thermal or compression injury. This study confirms a strong link to sepsis, multiple organ failure, and slow ventilatory wean. The requirement for a precise neurophysiological diagnosis of critical illness polyneuropathy needs to be balanced with technical considerations and the likelihood of alternative diagnoses.

Critical illness polyneuropathy (CIP) is an underreported condition, particularly in the context of severe burn injuries. It is a diffuse neuropathy that causes pure axonal damage predominantly in motor neurons, resulting in profound neuromuscular flaccid weakness in limbs and in severe cases, involves the diaphragm.^1^ The incidence reported is variable and ranges from as low as 2%^2^ to as high as 29%.^3^ The associated mortality and long-term sequelae demands improved awareness.

Despite documentation in the literature as early as 1971 by Henderson et al,^4^ the cause of this condition remains poorly understood. It is often anticipated in intensive care patients who have had sepsis, multiorgan failure and difficult weans from mechanical ventilation and intensive care unit (ICU) treatment. The pathophysiology of the condition has been speculated for several decades. Metabolic derangements, inflammatory mediated proteolysis, endotoxins, mitochondrial dysfunction, and hormonal deregulation have all been suggested.^5^ Men tend to develop CIP twice as often as women and patients 50 years or older are more susceptible. As well, electrical burns, full-thickness burns, and burns greater than 20% total body surface area (TBSA) have a higher prevalence.^4^,^6^ The use of steroids and neurotoxic agents such as neuromuscular blocking agents and certain antibiotics have been implicated with its role in myopathic changes.^7^,^8^ This may compound the muscular deconditioning caused by axonal involvement in burn patients with CIP.

Mortality rates in these patients may be 3 times higher than those without CIP.^9^ There are strong associations with prolonged duration on mechanical ventilation, lengthy ICU stays, and rehabilitation.^10^ There is significant functional impairment and reduction in overall quality of life in survivors. The severity of sequelae tends to correspond to the severity of sepsis and multiorgan failure. Complete paresis in the extremity does not always equate to poor long-term neurological outcome. This makes predicting recovery from initial presentations difficult.^11^

A review of the current literature on critical illness polyneuropathy in burn patients is performed. In most studies, a broad range of neurological findings, ranging from mononeuritis multiplex to diffuse distal axonal neuropathy, have been recorded. This study is directed toward further defining the cause and outcome of CIP after major thermal injuries.

## METHODS

The records of burn patients presenting to our burn unit between 2004 and 2009 are reviewed. This is a retrospective review of all severe burn patients who exhibited symptoms and signs of critical illness neuropathy and were suspected of the condition. Data such as percentage TBSA, burn cause, neurological deficits, and involvement of burnt or unburnt skin are collected. Other factors incorporated are complications in ICU and the use of neurotoxic agents. The number of days on the ventilator, the number of days spent in the ICU, and the length of hospital stay are shown on Table 1.

## RESULTS

During the 5-year period, there were 7 burn patients in total that manifested abnormal neurological findings. All were male, aged between 17 and 43, sustaining injuries from flame burns. The mean TBSA was 46%. A neuromuscular blocking agent like vecuronium was used in half of the cases. None had preexisting neurological conditions. Five patients had involvement of nerves that were away from the site of burn injury. Neurological presentation was varied, ranging from bilateral hand weakness, foot drop to involvement of proximal musculature (Table 1). Sensory pathologies were also observed. Upper and lower limbs appear to be affected equally. Six patients had hand involvement, exhibiting weakness. Out of the 5 with leg involvement, 4 had weakness manifesting below the knee. Only one had sensory changes associated with neuropathic pain.

Neurological examinations were difficult in most cases because of pain, anxiety, and heavy dressings. In patient 2, the global hypertonia, hyperreflexia, and marked clonus present in the lower limbs were initially attributed to a cerebral event or a metabolic or septic encephalopathy. However, computed tomography of the brain, septic, and metabolic screens returned normal.

Computed tomography was performed in 3 cases and no abnormalities were detected in the brain. An electroencephalogram and electromyogram were performed on 1 patient and returned with negative findings. This same patient had a serum electrophoretogram study carried out with a pattern consistent with acute activity on chronic inflammation. Magnetic resonance imaging of the brain was performed in 1 patient, and it was a normal study. This patient also had a nerve conduction study, which was suggestive of bilateral median nerve neuropathy. Out of the remaining 6 patients, 1 refused to consent for the test, 3 were not considered because of the areas of burnt skin involved and the heavy bandaging in place. The neurologists decided against performing the study in the remaining two, as there was no therapeutic gain.

Duration of stay was lengthy. Average hospital stay was 85 days, and the average time spent on the ventilator is 42 days. Intensive care complications are presented in Table 2. Sepsis was present in all 7 patients. There was no mortality in this study. One patient discharged against medical advice. Five were discharged to a nearby centre for further rehabilitation. On discharge, most had good recovery in the lower limbs. Hand function tended to recover poorly requiring protracted intensive hand therapy. Ongoing weak pincer and grip strength persisted at the 6-month to 1-year follow-up.

## DISCUSSION

This study highlights that neuromuscular degeneration is an unusual but not a rare complication in burn patients. It goes by varied names which serve to highlight the mysteries of the condition that remain largely unknown because of the complex nature of neurological pathophysiology in response to systemic insults.^12^ This can explain the varied neurological presentations observed in our study. In burn patients, diagnoses of mononeuritis multiplex, generalised axonal polyneuropathy, isolated mononeuropathy, and radiculopathy have all been documented in the literature. These conditions are postulated to be linked to the systemic inflammatory response that occurs in large burns.^2^,^6^,^13^

One patient had bilateral foot drop, which could not be explained by direct thermal injury or the resultant scarring and contracture. It is known that nerve compression from poor positioning or tight bandaging produces a transient neurapraxia without the lingering sequelae observed in CIP.^13^ One of the conclusions that can be drawn is the possibility of an underlying neuromuscular pathology underpinning the clinical weakness rather than entrapment neuropathy involving superficial nerves occurring at the site of the burn.^13^

The presence of prolonged sepsis and multiple organ failure observed in all 7 patients confirmed a strong link to CIP. Six had difficulties weaning from the ventilator. It is linked to higher mortality rates, and survivors face lengthy rehabilitation and reduced quality of life.^14^,^15^ We were not able to discern a predilection toward upper or lower limb involvement, but some studies have shown weakness in the lower extremities to be more prominent.^14^ This may be related to the length-dependent recovery of longer nerves. It was observed that all 5 patients had poor recovery of hand function, which may be explained by the density of motor units supplying the intrinsic muscles of the hand responsible for fine motor function. This significantly affected their quality of life and impeded their return to work and daily function.

All patients in our study were involved in flame burns. Electrical injuries have traditionally been associated with an increased risk of critical illness neuropathy.^6^,^13^,^16^ However, incomplete combustion of fire produces a considerable amount of carbon monoxide, which has been historically shown to cause central nervous system complications.^17^,^18^,^19^ In acute carbon monoxide poisoning, abnormalities in nerve conduction studies and electromyograms of lower extremities have been reported.^20^,^21^ Also, the release of chemicals such as n-hexane and benzene from petrol combustion has been linked to a variety of delayed neurological deficits such as symmetrical leg weakness, peripheral neuritis, and paraesthesia.^22^,^23^ Further studies are required to examine the neurotoxic effects of certain compounds produced from fuel combustion.

Reduction in action potential amplitude and conduction delay is an early sign of neuropathy that can occur well before the onset of clinical signs.^15^,^24^ Electromyographic studies and nerve conduction tests are often carried out with the aim of narrowing down the location and define the nature of the deficit. These tests also enable neurologists to differentiate between myopathic and neuropathic processes.^8^ However, some authors have suggested that clinical findings are sufficient evidence to diagnose CIP and as yet, there are no uniform diagnostic criteria.^25^ In addition, these tests were not always possible in clinical practice as demonstrated in our study. The ICU environment is not very conducive to nerve conduction testing due to the hostile background noise which interferes with accurate recording of nerve and muscle responses.^26^ Our efforts were further hampered by the presence of tissue oedema, joint contractures, pain and inadequate exposure from burn dressings to facilitate a thorough neurological assessment, and accurate nerve conduction testing. Patients were also reluctant to participate because of the discomfort caused by the electrodes especially when burnt skin is involved.

There is clearly a need for a simple and effective method of assessing weakness related to CIP. Ali et al^27^ suggested the use of a handheld dynamometer as a way of assessing handgrip strength which was lower in patients with CIP. It has been suggested that upper extremity involvement tends to have a poorer outcome.^14^ This technique could facilitate early detection of the condition and commencement of intense physiotherapy and occupational therapy input.^28^ However, the clinical feasibility and accuracy of this technique require further investigation.

## CONCLUSION

Our study confirms the strong link of CIP to systemic inflammatory response syndrome leading to sepsis and multi-organ failure. It highlights burns patients as a unique group of patients, as there are many intrinsic technical challenges that clinicians face in the diagnostic process. In many patients, it is often difficult to differentiate a central and peripheral neurological cause. This poses a clinical and electrodiagnostic challenge, which may be worthwhile pursuing if technical difficulties are considered and balanced with the possibility of alternative diagnoses to determine the urgency of the need for neurophysiological studies.

## Figures and Tables

**Table 1:** Summary of patients*

	Case	Clinical Findings	Site of Burns	Imaging and Studies	Days on Ventilator	Days in ICU	Hospital Stay, days	Neurotoxic Agents
1	43 years, M, flame (LPG)	Bilateral below knee weakness, hands weakness, glove and stocking sensory changes	53% PTB face, abdomen, both upper arms, thighs	EPG: acute on chronic inflammatory changes; normal CT, EEG, EMG	73	79	136	NMBA, gentamicin
2	38 years, M, flame (spirits)	Bilateral leg hypertonicity (L>R) with weakness, poor left hand power, confusional states	33% PTB face, neck, trunk, back and both upper arms	Normal CT	15	17	29	NMBA
3	25 years, M, flame (Molotov cocktail)	Bilateral foot drop (L>R), poor left hand grip strength	25% FTB R arm, L ear, back; 10% PTB face, L arm, flank, R foot	Normal CT	20	25	38	NMBA
4	36 years, M, flame (petrol)	Bilateral shoulder/ elbow/ hand weakness with wasting	35% FTB back, R arm, L leg; 30% PTB back, flank, L arm, R leg, buttocks	Normal CT	68	73	116	–
5	17 years, M, flame (petrol)	Bilateral wrist/ hand weakness, weak bilateral hip/ knee flexion	70% FTB both upper arms, thighs, anterior chest, face, neck	–	75	82	108	–
6	17 years, M, flame (spirits)	Right foot drop	30% PTB face, arms, both legs	–	16	21	32 (self-discharged)	gentamicin
7	63 years, M, flame (spirits)	Right shoulder, bilateral hands	23% FTB face, forearms, chest; 10% PTB chest, L thigh	NCS: bilateral median nerve neuropathy; normal MRI	12	14	36	–

*CT indicates computed tomography; EEG, electroencephalogram; EMG, electromyogram; EPG, electrophoretogram; FTB = full-thickness burn; ICU, intensive care unit; L, left; LPG, liquefied petroleum gas; M, male; MRI, magnetic resonance imaging; NMBA = neuromuscular blocking agent; NCS = nerve conduction study; PTB = partial-thickness burn; R = right.

**Table 2:** Complications in intensive care unit

	Patients, n	Total, %
Sepsis	7	100
Slow ventilatory wean	6	86
Respiratory failure	3	43
Renal failure	3	43
Coagulopathy	5	71
Encephalopathy	1	14
